# Supplementary material for: Modeling glioblastoma heterogeneity as a dynamic network of cell states
Source: Mol Syst Biol. 2021 Sep 16;17(9):e10105. doi: 10.15252/msb.202010105 (PMC8444284; doi:10.15252/msb.202010105)
Supplement: Supplementary file 6 — Source Data for Figure 5 [file MSB-17-e10105-s004.zip › Figure5A_sourcedata/GSEA_3071/hallmarks_stateV.GseaPreranked.1623742546329/HALLMARK_COMPLEMENT.html]

Details for gene set HALLMARK\_COMPLEMENT[GSEA]

|  || Dataset | state43071 |
| Phenotype | NoPhenotypeAvailable |
| Upregulated in class | na\_neg |
| GeneSet | HALLMARK\_COMPLEMENT |
| Enrichment Score (ES) | -0.43287358 |
| Normalized Enrichment Score (NES) | -2.2569392 |
| Nominal p-value | 0.0 |
| FDR q-value | 0.0 |
| FWER p-Value | 0.0 |
Table: GSEA Results Summary

  

Fig 1: Enrichment plot: HALLMARK\_COMPLEMENT      
 Profile of the Running ES Score & Positions of GeneSet Members on the Rank Ordered List

  

| PROBE | GENE SYMBOL | GENE\_TITLE | RANK IN GENE LIST | RANK METRIC SCORE | RUNNING ES | CORE ENRICHMENT || 1 | CDH13 |  |  | 185 | 1.204 | -0.0121 | No |
| 2 | PDGFB |  |  | 283 | 1.080 | -0.0098 | No |
| 3 | DUSP6 |  |  | 408 | 0.944 | -0.0152 | No |
| 4 | CLU |  |  | 464 | 0.900 | -0.0083 | No |
| 5 | PLAT |  |  | 651 | 0.773 | -0.0289 | No |
| 6 | GNAI2 |  |  | 845 | 0.681 | -0.0527 | No |
| 7 | PRCP |  |  | 983 | 0.626 | -0.0668 | No |
| 8 | PDP1 |  |  | 1006 | 0.619 | -0.0590 | No |
| 9 | CASP3 |  |  | 1057 | 0.605 | -0.0569 | No |
| 10 | TIMP2 |  |  | 1422 | 0.498 | -0.1170 | No |
| 11 | GNB4 |  |  | 1505 | 0.479 | -0.1234 | No |
| 12 | MT3 |  |  | 1531 | 0.474 | -0.1190 | No |
| 13 | RAF1 |  |  | 1578 | 0.464 | -0.1188 | No |
| 14 | ZEB1 |  |  | 1605 | 0.459 | -0.1149 | No |
| 15 | C1R |  |  | 1696 | 0.437 | -0.1237 | No |
| 16 | CALM3 |  |  | 1710 | 0.435 | -0.1177 | No |
| 17 | RNF4 |  |  | 1726 | 0.432 | -0.1122 | No |
| 18 | RBSN |  |  | 1729 | 0.431 | -0.1043 | No |
| 19 | KIF2A |  |  | 1857 | 0.409 | -0.1207 | No |
| 20 | GPD2 |  |  | 2222 | 0.359 | -0.1834 | No |
| 21 | ADAM9 |  |  | 2228 | 0.359 | -0.1774 | No |
| 22 | PPP2CB |  |  | 2237 | 0.358 | -0.1720 | No |
| 23 | MSRB1 |  |  | 2357 | 0.341 | -0.1882 | No |
| 24 | CTSV |  |  | 2541 | 0.317 | -0.2171 | No |
| 25 | GNG2 |  |  | 2787 | 0.291 | -0.2584 | No |
| 26 | GCA |  |  | 2870 | 0.283 | -0.2686 | No |
| 27 | MMP15 |  |  | 2885 | 0.282 | -0.2658 | No |
| 28 | CDK5R1 |  |  | 2919 | 0.277 | -0.2668 | No |
| 29 | PPP4C |  |  | 3063 | 0.263 | -0.2891 | No |
| 30 | CALM1 |  |  | 3080 | 0.261 | -0.2871 | No |
| 31 | PIK3CA |  |  | 3120 | 0.258 | -0.2895 | No |
| 32 | USP15 |  |  | 3388 | -0.279 | -0.3352 | No |
| 33 | HSPA1A |  |  | 3494 | -0.299 | -0.3496 | No |
| 34 | LTA4H |  |  | 3584 | -0.312 | -0.3606 | No |
| 35 | ATOX1 |  |  | 3627 | -0.320 | -0.3624 | No |
| 36 | CASP1 |  |  | 3648 | -0.323 | -0.3600 | No |
| 37 | DOCK9 |  |  | 3658 | -0.324 | -0.3554 | No |
| 38 | TNFAIP3 |  |  | 3691 | -0.330 | -0.3552 | No |
| 39 | CTSO |  |  | 3774 | -0.344 | -0.3642 | No |
| 40 | MAFF |  |  | 3849 | -0.359 | -0.3714 | No |
| 41 | GZMB |  |  | 3906 | -0.368 | -0.3750 | No |
| 42 | CPQ |  |  | 3933 | -0.374 | -0.3727 | No |
| 43 | PCLO |  |  | 3963 | -0.380 | -0.3709 | No |
| 44 | GNAI3 |  |  | 3965 | -0.381 | -0.3637 | No |
| 45 | GATA3 |  |  | 4015 | -0.392 | -0.3655 | No |
| 46 | CD59 |  |  | 4024 | -0.394 | -0.3594 | No |
| 47 | CTSS |  |  | 4043 | -0.398 | -0.3551 | No |
| 48 | LIPA |  |  | 4436 | -0.516 | -0.4202 | No |
| 49 | PSMB9 |  |  | 4503 | -0.543 | -0.4224 | Yes |
| 50 | EHD1 |  |  | 4533 | -0.555 | -0.4171 | Yes |
| 51 | CASP4 |  |  | 4551 | -0.565 | -0.4094 | Yes |
| 52 | ANXA5 |  |  | 4616 | -0.601 | -0.4101 | Yes |
| 53 | HSPA5 |  |  | 4714 | -0.652 | -0.4160 | Yes |
| 54 | DOCK10 |  |  | 4777 | -0.696 | -0.4144 | Yes |
| 55 | DYRK2 |  |  | 4782 | -0.699 | -0.4016 | Yes |
| 56 | PIM1 |  |  | 4783 | -0.699 | -0.3880 | Yes |
| 57 | CTSL |  |  | 4785 | -0.701 | -0.3746 | Yes |
| 58 | S100A13 |  |  | 4791 | -0.705 | -0.3619 | Yes |
| 59 | DUSP5 |  |  | 4796 | -0.707 | -0.3490 | Yes |
| 60 | CD55 |  |  | 4834 | -0.742 | -0.3417 | Yes |
| 61 | ME1 |  |  | 4982 | -0.899 | -0.3524 | Yes |
| 62 | CTSB |  |  | 5007 | -0.948 | -0.3386 | Yes |
| 63 | LGALS3 |  |  | 5039 | -1.017 | -0.3249 | Yes |
| 64 | CEBPB |  |  | 5076 | -1.088 | -0.3107 | Yes |
| 65 | IRF1 |  |  | 5100 | -1.155 | -0.2927 | Yes |
| 66 | CSRP1 |  |  | 5130 | -1.243 | -0.2742 | Yes |
| 67 | TIMP1 |  |  | 5134 | -1.251 | -0.2505 | Yes |
| 68 | MMP14 |  |  | 5146 | -1.297 | -0.2274 | Yes |
| 69 | SERPINE1 |  |  | 5161 | -1.377 | -0.2034 | Yes |
| 70 | PLAUR |  |  | 5185 | -1.479 | -0.1792 | Yes |
| 71 | COL4A2 |  |  | 5198 | -1.545 | -0.1515 | Yes |
| 72 | LRP1 |  |  | 5234 | -1.801 | -0.1233 | Yes |
| 73 | F3 |  |  | 5242 | -1.927 | -0.0873 | Yes |
| 74 | CFH |  |  | 5257 | -2.141 | -0.0485 | Yes |
| 75 | FN1 |  |  | 5287 | -2.866 | 0.0015 | Yes |
Table: GSEA details [plain text format]

  

Fig 2: HALLMARK\_COMPLEMENT: Random ES distribution      
 Gene set null distribution of ES for **HALLMARK\_COMPLEMENT**
